# Draft Genome Sequences of 50 Methicillin-Resistant *Staphylococcus aureus* Sequence Type 5 Isolates Obtained from a U.S. Hospital

**DOI:** 10.1128/genomeA.01083-17

**Published:** 2017-11-02

**Authors:** Samantha J. Hau, Darrell O. Bayles, David P. Alt, Tracy L. Nicholson

**Affiliations:** a Department of Veterinary Diagnostic and Production Animal Medicine, College of Veterinary Medicine, Iowa State University, Ames, Iowa, USA; b National Animal Disease Center, Agriculture Research Service, USDA, Ames, Iowa, USA

## Abstract

Methicillin-resistant *Staphylococcus aureus* (MRSA) can be a commensal or pathogen in humans. Pathogenicity and disease are related to the acquisition of mobile genetic elements encoding virulence and antimicrobial resistance genes. Here, we report draft genome sequences for 50 clinical MRSA isolates from humans with MRSA-related disease.

## GENOME ANNOUNCEMENT

Methicillin-resistant *Staphylococcus aureus* (MRSA) was first isolated in 1961 (1). MRSA rapidly became widespread in the hospital setting and remained contained there until the late 1990s, when isolates began infecting patients with no known risk factors for hospital-acquired MRSA (HA-MRSA) (2). This subset of MRSA isolates was termed community-acquired MRSA (CA-MRSA). The third group of isolates are acquired by contact with livestock species and are called livestock-associated MRSA (LA-MRSA). Specific lineages of *S. aureus* predominate within each subset, and these lineages are defined by their multilocus sequence type (ST). *S. aureus* lineages are defined by differing characteristics that allow them to possess distinct niches. The ST5 lineage, specifically, is a widespread and successful lineage of HA-MRSA (3). This is primarily attributed to the capacity of this lineage to acquire mobile genetic elements encoding virulence factors and antimicrobial resistance genes (3).

Here, we report the generation of 50 draft genome sequences from MRSA ST5 isolates obtained from the hospital at the University of California, Irvine (4). Isolates sequenced, listed in Table 1, were from patients who had MRSA-related disease and no known livestock exposure. Minimal patient history was available, including source information, making HA- and CA-MRSA indistinguishable. The isolates were grown in trypticase soy broth (BD Biosciences, Sparks, MD), and the High Pure Template preparation kit (Roche Applied Science, Indianapolis, IN) was used to isolate total genomic DNA.

**TABLE 1:** Assembled draft genome sequences obtained from this project

Isolate name	Avg coverage	No. of contigs	GenBank accession no.
UCI 1	73.13	89	LKYS00000000
UCI 2	51.15	162	LKYT00000000
UCI 4	69.93	137	LKYV00000000
UCI 5	83.47	105	LKYW00000000
UCI 6	59.19	111	LKYX00000000
UCI 7	51.04	158	LKYY00000000
UCI 8	53.59	120	LKYZ00000000
UCI 10	50.26	125	LKZB00000000
UCI 12	61.68	167	LKZD00000000
UCI 13	46.01	144	LKZE00000000
UCI 14	43.15	183	LKZF00000000
UCI 15	46.20	123	LKZG00000000
UCI 16	83.09	117	LKZH00000000
UCI 17	83.32	140	LKZI00000000
UCI 18	55.44	205	LKZJ00000000
UCI 20	55.04	201	LKZL00000000
UCI 22	59.19	148	LKZN00000000
UCI 23	38.77	311	LKZO00000000
UCI 25	80.63	125	LKZQ00000000
UCI 26	71.95	114	LKZR00000000
UCI 28	48.98	145	LKZT00000000
UCI 29	65.78	247	LKZU00000000
UCI 30	66.07	148	LKZV00000000
UCI 31	56.88	219	LKZW00000000
UCI 32	43.65	269	LKZX00000000
UCI 33	43.35	242	LKZY00000000
UCI 34	49.12	224	LKZZ00000000
UCI 35	46.42	111	LLAA00000000
UCI 36	46.00	246	LLAB00000000
UCI 37	37.53	264	LLAC00000000
UCI 38	53.12	227	LLAD00000000
UCI 39	83.79	107	LLAE00000000
UCI 40	44.79	171	LLAF00000000
UCI 41	50.09	175	LLAG00000000
UCI 42	54.66	160	LLAH00000000
UCI 44	60.57	140	LLAJ00000000
UCI 47	65.17	191	LLAM00000000
UCI 49	57.85	237	LLAO00000000
UCI 50	50.05	174	LLAP00000000
UCI 51	41.44	184	LLAQ00000000
UCI 53	69.80	97	LLAS00000000
UCI 54	49.38	138	LLAT00000000
UCI 55	80.91	156	LLAU00000000
UCI 57	80.99	87	LLAW00000000
UCI 58	79.17	112	LLAX00000000
UCI 59	82.31	132	LLAY00000000
UCI 60	49.91	231	LLAZ00000000
UCI 61	66.52	178	LLBA00000000
UCI 62	61.44	131	LLBB00000000
UCI 63	54.37	96	LLBC00000000

Draft genome sequences were produced using the Illumina MiSeq platform. Indexed libraries were produced with the Nextera XT DNA sample preparation and index kit (Illumina, San Diego, CA). Sequencing employed the MiSeq v2 500 Cycle reagent kit (Illumina) and generated 2 × 250-bp paired-end reads.

Sequence reads were assembled into draft genomes using MIRA v4.0.2 (http://mira-assembler.sourceforge.net/docs/DefinitiveGuideToMIRA.html). The average coverage for each isolate is listed in Table 1. For retention in the assembly, the contigs were required to be >1,500 bp and have a coverage with at least two-thirds the average coverage of the genome. When repetitive elements were identified during assembly, the contig was required to be >2,000 bp to be included in the assembly.

### Accession number(s).

The assembled draft genome sequences obtained from this project were deposited into DDBJ/ENA/GenBank with the accession numbers listed in Table 1.
